# In vitro contact guidance of glioblastoma cells on metallic biomaterials

**DOI:** 10.1007/s10856-021-06503-z

**Published:** 2021-03-29

**Authors:** B. Uzer-Yilmaz

**Affiliations:** Department of Mechanical Engineering, Abdullah Gül University, 38080 Kayseri, Turkey

## Abstract

Cancer cells’ ability to sense their microenvironment and interpret these signals for the regulation of directional adhesion plays crucial role in cancer invasion. Furthermore, given the established influence of mechanical properties of the substrate on cell behavior, the present study aims to elucidate the relationship between the contact guidance of glioblastoma cell (GBM) and evolution of microstructural and mechanical properties of the implants. SEM analyses of the specimens subjected to 5 and 25% of plastic strains revealed directional groove-like structures in micro and submicro-sizes, respectively. Microscale cytoplasmic protrusions of GBMs showed elongation favored along the grooves created via deformation markings on 5% deformed sample. Whereas filopodia, submicro-sized protrusions facilitating cancer invasion, elongated in the direction perpendicular to the deformation markings on the 25% deformed sample, which might lead to easy and rapid retraction. Furthermore, number of cell attachment was 1.7-fold greater on 25% deformed sample, where these cells showed the greatest cellular aspect ratio. The directional attachment and contact guidance of GBMs was reported for the first time on metallic implants and these findings propose the idea that GBM response could be regulated by controlling the spacing of the deformation markings, namely the degree of plastic deformation. These findings can be applied in the design of cell-instructive implants for therapeutic purposes to suppress cancer dissemination.

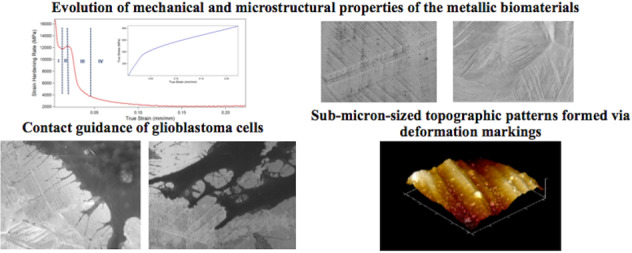

## Introduction

Metallic materials have been used as artificial substitutes, fixture apparatus or therapeutic appliance in numerous biomedical applications, such as hip replacements in orthopedics, stents in cardiovascular surgery, or archwires in orthodontics [1–3]. Austenitic stainless steel has been one of the most widely used alloys among metallic biomaterials because of its good corrosion resistance, biocompatibility, availability and ease of formability [1–3]. Moreover, it has good mechanical strength and ductility, owing to distinctive hardening response ascribed to activation of deformation twinning, which stems from low stacking fault energy (SFE) [4–9].

In these low SFE FCC metals, perfect dislocation can split into partial dislocations and form stacking faults on overlapping {111} planes, which would be the precursor of deformation twins or martensites [10]. Activation of either of these mechanisms depends strongly on grain size [6, 8]. Interestingly, martensite is induced primarily on the samples having coarse grains and deformation twinning becomes the governing mechanism as the grains get finer [3]. Furthermore, martensite transformation can depend on SFE, such that SFE lower than 18 mJm^−2^ favors direct transformation of γ-austenite (fcc) → ε-martensite (hcp) → α’**-**martensite (bcc) [11]. Hence, elucidating the highly complex hardening mechanism of low SFE FCC metals can play a crucial role in terms of predicting plastic deformation capability of implants during processing operations or service period.

Prior research on microstructure evolution of low SFE FCC metals has reported hardening response of these metals under tensile and compressive loadings [4, 5, 11–14]. High strain-hardening rate and ductility of these steels has been specifically attributed to activation of deformation twinning [4, 7, 15]. Martensite formation also contributes to hardening of the material, through the effect known as transformation induced plasticity (TRIP) [11]. Specifically, similar to grain boundaries, mechanical twin boundaries and martensite platelets inhibit motion of dislocations and reduce their mean free path [13]. As a result, these boundaries act as sites for dislocation pile-ups and storage, which lead to strengthening of the material [15, 16].

The aforementioned deformation mechanisms can be induced on implant via manufacturing processes like extrusion, rolling, drawing or forging [17]. Annealing process carried out to improve ductility after cold working may not perfectly recrystallize the metal, and can leave the material with these defects and anisotropic texture with preferred orientations [18]. These mechanisms can also be activated during implantation of the metal in the body. For instance, cardiovascular stents can be given as exemplars of inevitable formation of deformation markings on the metal surface upon severe plastic deformation taking place during dilatation of the stent inside the artery [19].

Recently, the interaction of these deformation mechanisms with cancer and healthy cells has been reported [2, 3, 20–23]. Topographical reflections of these mechanisms, named as surface relief, result in micro- to nanoscale intricate patterns, i.e., grooves or channels. These patterns increase surface-to-volume ratio and provide accessible sites for the deposition of focal adhesion molecules, which successively enhance cell adhesion [2, 20–22]. Moreover, they lead to increased surface roughness, wettability, and free energy [22].

A study by Matsugaki et al. has further revealed the directional cell adhesion along deformation markings, where actin stress fibers of osteoblasts elongated along and vinculin deposited within the steps generated via slip traces [20]. This means that, oriented structure of the deformation markings in each crystal mimicking the directional characteristics of 3D extra cellular matrix (ECM) may be helpful to achieve anisotropic morphogenesis of the bone tissue [20, 24]. Eventually, this would provide a more effective cell response and enhance the success of the implantation [25].

Directional adhesion behavior could also be exploited by cancer cells, where topographical cues can be facilitated for directional migration necessitated in cancer invasion [26]. This behavior, named as contact guidance, requires coordinated movement of protrusive activities, i.e., filopodia and lamellipodia, along these cues [24, 27, 28]. Lamellipodia, highly branched actin filaments, have been the focus of research in understanding how cellular processes work, until substrate exploring role of filopodia, submicro-scale unidirectional and parallel actin filaments, was explained in 1976 [29]. Such that, they can serve as cells’ antennae and sense topography in pursue of physical cues by assembling specialized adhesion complexes [27] and allow guidance of cells along the surface patterns [29]. Hence, analyzing the contact guidance of cancer filopodia on substrates with topographic cues could be of importance in understanding and controlling invasion of cancer.

Contact guidance of cancer cells has been investigated by using synthetic or natural materials commonly based on hydrogels, which are designed to mimic aligned topography of tumor microenvironment [26, 30–33]. Furthermore, given the established influence of mechanical properties of the substrate on cancer cell invasiveness [26, 33], effects of material stiffness on cell response were also investigated [31, 34]. Observed contact guidance and cell remodeling to physical and mechanical cues can indicate that cancer cell behavior can be regulated via these modifications in material. These studies have paved the way for understanding the cancer invasion behavior and identifying therapeutic targets.

Accordingly, the present study purports to shed light on the influence of evolution of microstructural and mechanical properties of metallic biomaterials on contact guidance of glioblastoma cells (hereafter named as GBMs). GBM is the most malignant, aggressive, and a currently incurable type of a brain tumor, where median survival of patients is approximately 15 months after diagnosis [30, 35, 36]. Diffuse invasion is one of the mechanisms GBMs hold to disseminate into neighboring tissues, and therefore continues to be the major subject of numerous cancer studies [35, 37, 38].

Clinical observations showed that during invasion GBMs migrate as single cells along white matter tracts, which have aligned nanoscale topography [31]. Noting the fact that directional migration is necessitated in GBM invasion, use of metallic biomaterials mimicking oriented topography of GBM microenvironment could offer insight into complex invasion mechanism of cancer cells. It should be noted that, contact guidance of cancer cells on ultrastiff materials such as metallic biomaterials (typically having elastic moduli of the order of tens of gigapascal or higher) has not been thoroughly analyzed yet. Noting the promising properties and potential therapeutic applications of metallic biomaterials in numerous biomedical fields, their effects on microenvironment and cell response warrant further investigation. The findings could be utilized to design implants for therapeutic purposes for cancer patients, where effective control of cancer invasion can be achieved.

## Materials and methods

The material subjected to the experiments in the study was austenitic stainless steel in the grade of 304. In order to activate deformation markings and form groove-like structures in different length scales on the material surface, specimens with the thickness of 1mm were subjected to tensile test at ambient temperature with strain rate of 10^−4^ s^−1^ and up to the engineering strains of 5 and 25% (samples hereafter named as 5 and 25%).

In order to eliminate topographical stimuli on cells, two sets of samples were prepared, where only one of them was polished post-mortem down to 1 μm with diamond abrasives (hereafter named as polished-samples) Then, these samples were etched at ambient temperature for 3 min in a solution of 10 ml nitric acid, 30 ml hydrochloric acid and 30 ml distilled water to reveal the activated mechanisms [39]. The microstructural evolution of the samples was observed via optical microscope (OM) in DIC mode and field-emission scanning electron microscope (SEM) with in-lens SE (secondary electron) detector under an accelerating voltage of 10 kV.

Chemical compositions of the samples were quantified via X-ray fluorescence (XRF) and the results have been used to calculate the stacking fault energy (SFE) with implementing the data in the formula proposed by Schramm and Reed [40]. Characterization of crystal structures of the samples were carried out with X-ray diffraction (XRD) operating at 45 kV and 40 mA with CuKα radiation, and step size of 0.02°.

Evolution of the mechanical properties of the samples has also been investigated. Hardening response of the materials was analyzed by taking derivative of true stress data of the 25% sample with respect to the true strain. The calculated strain-hardening rate (SHR) has been plotted versus true strain. Nanohardness and indentation depths of samples were analyzed via the nanoindentation technique, where a nano indenter equipped with diamond Berkovich tip was used. Indentations were carried out in load-control mode up to maximum load of 100 mN with constant 10 mN/s of loading rate and holding time of 5 s at maximum load.

Topography and spacing of the deformation markings were analyzed via atomic force microscopy (AFM). The analysis was carried out with tapping mode in air utilizing a phosphorus doped silicon cantilever with a rotated tip (radius of 8 nm) and linear scanning rate was of 1 Hz (1 line/s). Initially a 50 μm by 50 μm area was scanned. Average surface roughness (R_a_) of the samples are reported by taking the average R_a_ of at least four different regions with the aforementioned dimensions. Next, a narrower region within this area was selected and scanned, where more organized and measurable deformation markings were present. Obtained 2D and 3D images were analyzed and furthermore height and width of the grooves created by the deformation markings were measured (from at least three different regions) by creating a line profile traversing over these deformation mechanisms.

Next, cell attachment and spreading behavior was analyzed through culturing U373 GBMs on steel samples. Cells obtained from American Type Culture Collection (ATCC) were grown in standard culture conditions in DMEM supplemented with 10% FBS and 1% Penicillin-Streptomycin (Gibco). Next, cells were engineered to express GFP via retroviral transduction. Accordingly, cells were transduced with pBabeGFP viral particles in medium containing protamine sulfate (10mg/ml) at a multiplicity of infection (MOI) of 1. Meanwhile, metal samples were sterilized with an autoclave and placed into 24-well plate (Costar). Next, GBMs were added on each sample (1 × 10^5^ cells/well) with 1 ml volume of growth medium and were incubated at 37 °C. Upon 24 h of incubation period cells were fixed with 2.5% glutaraldehyde in PBS for 2 h. After fixation, cells were dehydrated with 70, 80, 90, and 100% ethanol solutions successively and dried in air. In order to analyze cytoplasmic protrusions in detail, samples were sputter-coated with 5 nm thickness of gold to mitigate charging problem and analyzed with scanning electron microscope (SEM) under an accelerating voltage of 3 kV and utilizing secondary electron (SE) detector. Filopodia orientation was examined using SEM images. ImageJ software was utilized for measuring the orientation relative to (1) deformation markings on the 25% sample and (2) edges of cells on the polished-25% sample. The measurements are reported as the average angle of at least 20 filopodia on each sample.

Immunofluorescence technique was utilized to observe the effect of plastic deformation and surface roughness on GBMs’ morphology. Actin, vinculin and cell nuclei staining were carried out as described elsewhere [41]. Cell number and elongation behavior was quantified using ImageJ. Diameters of major and minor axis of ellipse fitted around the cells were measured and cell aspect ratio (AR) was calculated by taking ratio of major axis to minor axis. It should be noted that cells on the edge of the fluorescence images were excluded from both cell number and elongation analyses.

The reported results are given as mean ± standard deviation values. Statistical significance of the differences between the means was evaluated with one-way analysis of variance (ANOVA) followed by least-square difference (LSD) post hoc comparison tests. Non-parametric equivalents of these tests were also reported to support findings. A *p* value smaller than 0.05 was required for the rejection of null hypotheses (highest Type-I error level deemed acceptable was set at 0.05).

## Results

The elemental composition of the samples obtained via XRF is presented in Table 1. This data were used to determine the stacking fault energy of the alloy, which was calculated as 15.624 mJm^−2^ based on the formula proposed by Schramm and Reed [40]. This SFE value places the samples tested in the study into the group of FCC metals with low SFE [42].

**Table 1:** XRF results presenting chemical composition of the 304 stainless steel samples (in wt. %)

Ni	Cr	Mn	Mo	C	Si	P	S	Cu	Fe
8.314	18.414	1.044	0.091	0.029	0.369	0.022	0.023	0.416	Bal.

**Fig. 1:**
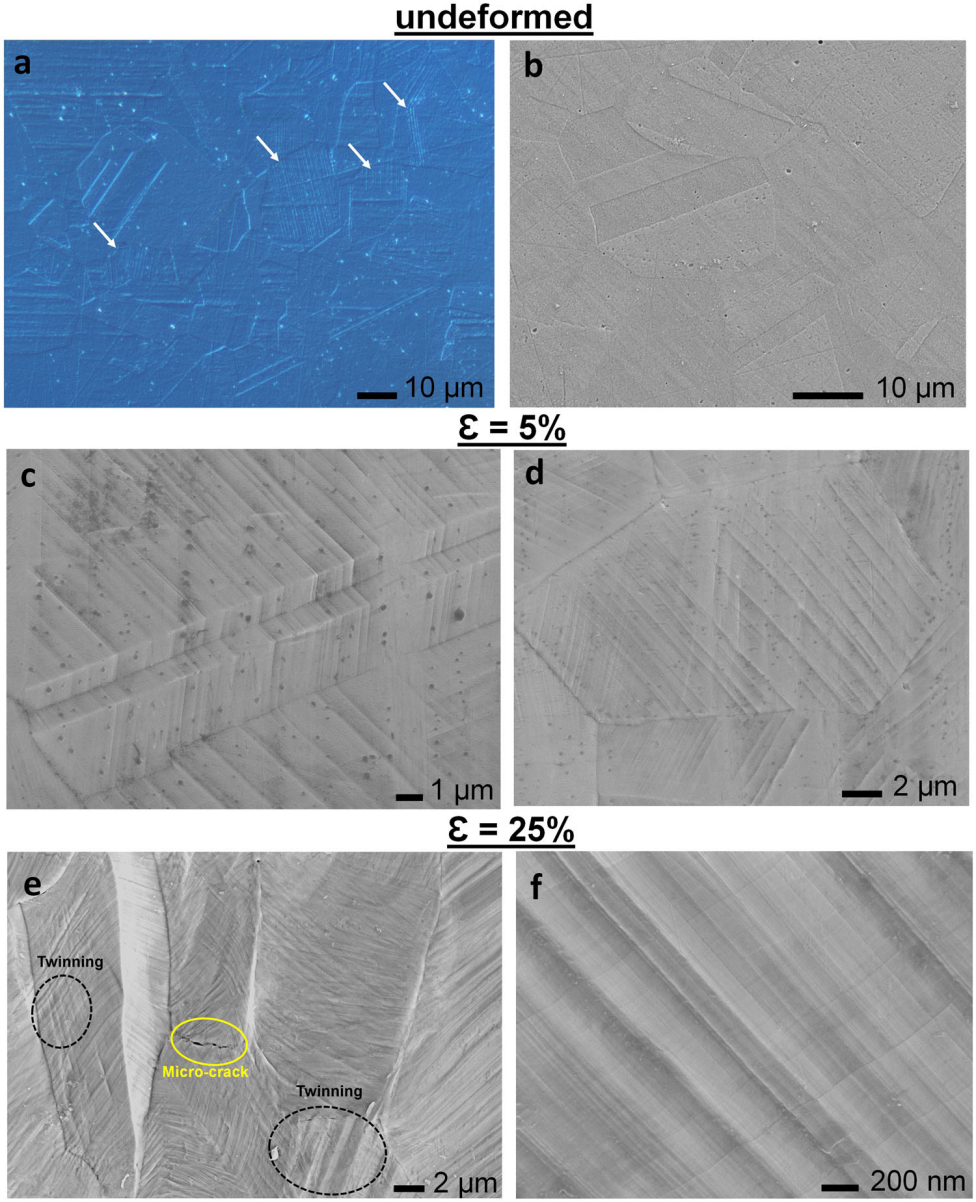
Plastic deformation induced microstructural evolution of stainless steel samples. **a** Optical microscope and **b** SEM image of undeformed sample present annealing twins. **c**, **d** SEM images of 5% deformed sample present annealing twin and deformation markings with microscale spacing. **e** Severely distorted topography of 25% deformed sample exhibits micro-crack and twinned regions. **f** Deformation markings with submicro-scale spacing on 25% sample. (Figure is more readable and visible on the web version rather than in print)

**Fig. 2:**
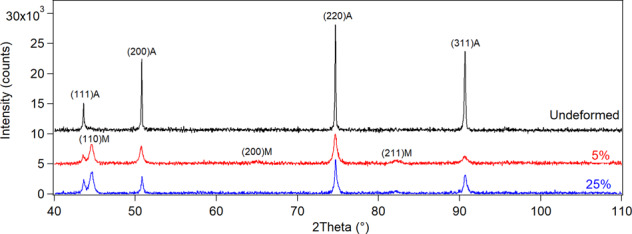
XRD spectra of the undeformed, 5%, and 25% deformed 304 stainless steel samples. Martensite peaks can be observed on deformed samples, whereas the undeformed sample presents fully austenitic crystal structure

Microstructures of the samples were analyzed via OM and SEM. OM images present annealing twins on the undeformed sample, as usually observed in low SFE austenitic steels [17]. However, etching of this sample has revealed localized deformations mechanisms, such as mechanical twins and/or martensite platelets as the arrows in Fig. 1a indicate. It should be mentioned that, these structures were not uniformly distributed and were observed in localized regions on the material in low amounts compared to the 5 and 25% samples. Moreover, XRD results of this sample present fully austenite structure, whereas, upon plastic deformation martensite peaks are detected on the deformed samples. It is also noteworthy that in parallel with plastic strain new martensite peaks were detected on 25% sample (Fig. 2).

SEM images of the deformed samples showed that the observed deformation markings on the 5% samples (Fig. 1c), believed to be slip bands, are straight in each grain and coplanar with the annealing twins. Moreover, in addition to these prominent slip markings, activation of secondary slip system can be observed in another set of {111} planes with much less density (Fig. 1d). The volume of these markings increases on the 25% sample, concomitant with the activation of multiple slip and twin systems. Figure 1e presents the microstructure of the 25% sample, where activated deformation twins intersect with other twin variants. Deformation markings of this sample show detectable curvatures and initiation of micro-crack was also observed on the grain boundary of the 25% sample (Fig. 1e).

SEM images of groove-like patterns created via deformation markings on the 5% (Fig. 1c, d) and the 25% (Fig. 1f) samples were analyzed and compared. It is noteworthy that as the plastic strain increases, the spacing of the deformation markings decreases from microscale to submicro-scale.

Surface topography analyses carried out via AFM presented R_a_ values of undeformed, 5%, and 25% samples, respectively as 17.08 ± 1.40 nm, 139 ± 5.97 nm and 447.25 ± 36.76 nm. Non-parametric Kruskal–Wallis tests reject the hypothesis that the means of all three groups are equal to each other (*p* < 0.01). One-way ANOVA and following multiple comparisons (LSD) suggest that mean values for each group is significantly different from all others at *p* < 0.001 or better level.

**Fig. 3:**
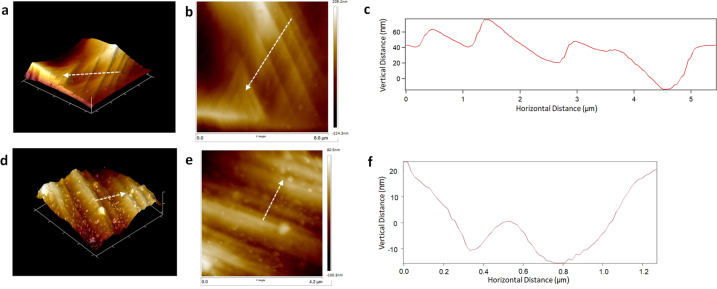
AFM analyses of plastic deformation induced surface relief and introduced deformation markings on deformed surfaces. Deformation markings with microscale spacing on the 5% sample were captured with (**a**) 3D and (**b**) height images; **c** Line profile presents vertical distance along the region traced with dashed arrow. Deformation markings with submicro-scale spacing were captured on the 25% sample with (**d**) 3D and (**e**) height images; **f** Line profile presents vertical distance along the region traced with dashed arrow

Surface texture obtained via deformation markings has also been analyzed via AFM. Figure 3a, d presents the formation of groove-like patterns on the topography of 5 and 25% samples, respectively, which are reflections of the deformation mechanisms on the surface. However, their depth and width differentiates as shown with line profile traversing over these grooves. Such that, the width and depth of the grooves created on the 5% sample was measured on average as 1.048 ± 0.22 μm and 28 ± 6.30 nm respectively. On the other hand the same dimensions were measured approximately as 319 ± 80 nm and 18 ± 4.67 nm on the 25% sample. Non-parametric Mann–Whitney *U* tests indicate that mean values of 5 and 25% groups are significantly different in terms of width (*p* < 0.005), whereas the difference appears to be non significant in terms of depth (*p* = 0.065).

**Fig. 4:**
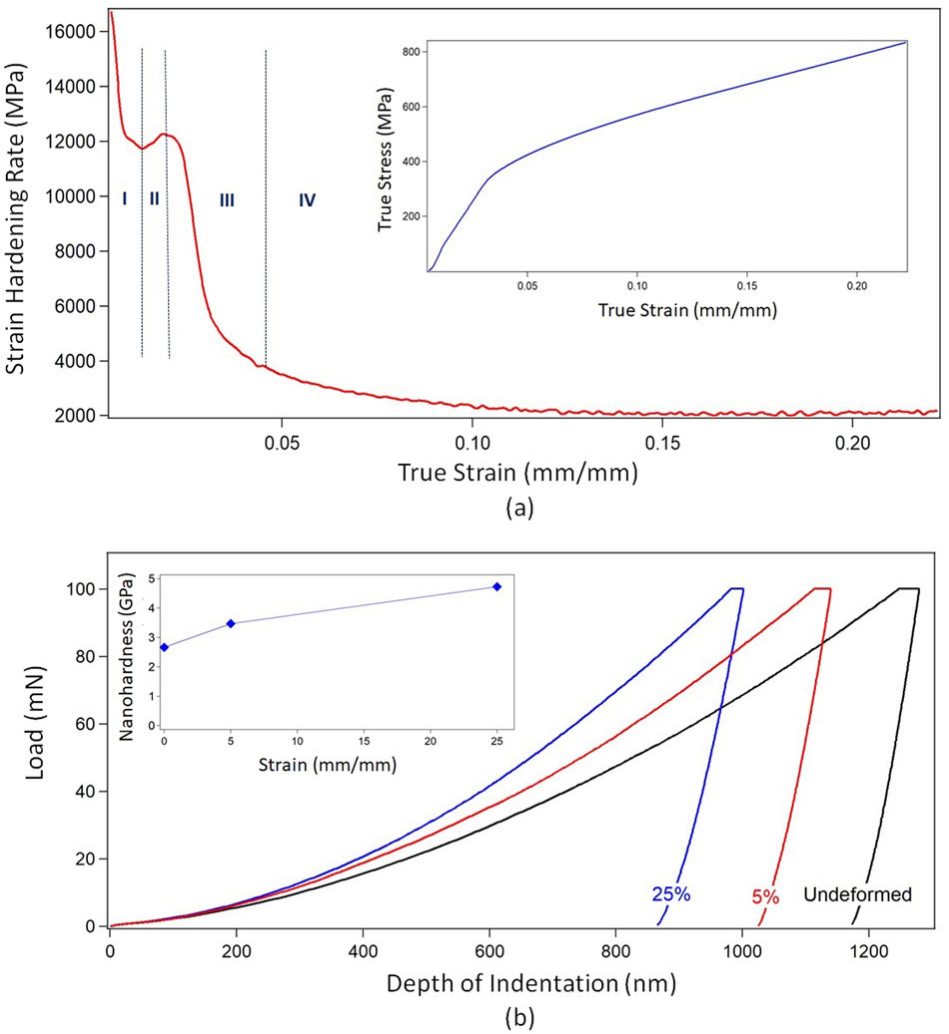
Evolution of mechanical properties of the deformed compared to undeformed (control) sample. The graphs present: **a** the strain-hardening rate versus true strain of 304 stainless steel specimens subjected to 25% of plastic strain. The inset graph shows the true stress-true strain of the sample. **b** Load vs. indentation depth curves of the undeformed and deformed samples obtained via nanoindentation test. The inset graph presents nanohardness vs. strain

In order to understand the relation between microstructural evolution and mechanical response of samples, strain-hardening rate (SHR) versus true strain curve of the 25% sample was plotted. As shown in the Fig. 4a, the hardening response of this sample presents multi-stage behavior, consisting of 4 distinct stages similar to the low SFE FCC metals [4, 7, 14, 43]. Decreasing SHR is observed in stage I in a similar way with stage III. Whereas stage II presents an increase of this rate, and in stage IV SHR is sustained at a constant level for a large strain range. The effect of plastic deformation on surface hardness was also analyzed via the nanoindentation technique. The load versus depth to indentation curves of the samples seem to present a shift towards lower indentation depths along with increasing plastic strain (Fig. 4b). In addition, average nanohardness values of undeformed, 5 and 25% samples were measured respectively as 2.67 ± 0.06, 3.47 ± 0.07 GPa and 4.73 ± 0.06 GPa (inset graph in Fig. 4b). Non-parametric Kruskal–Wallis test rejects the null hypothesis that all group means are equal (*p* < 0.05). Further, one-way ANOVA and following multiple comparisons (LSD) suggest that mean values for each group is significantly different from all others at *p* < 0.001 or better level.

**Fig. 5:**
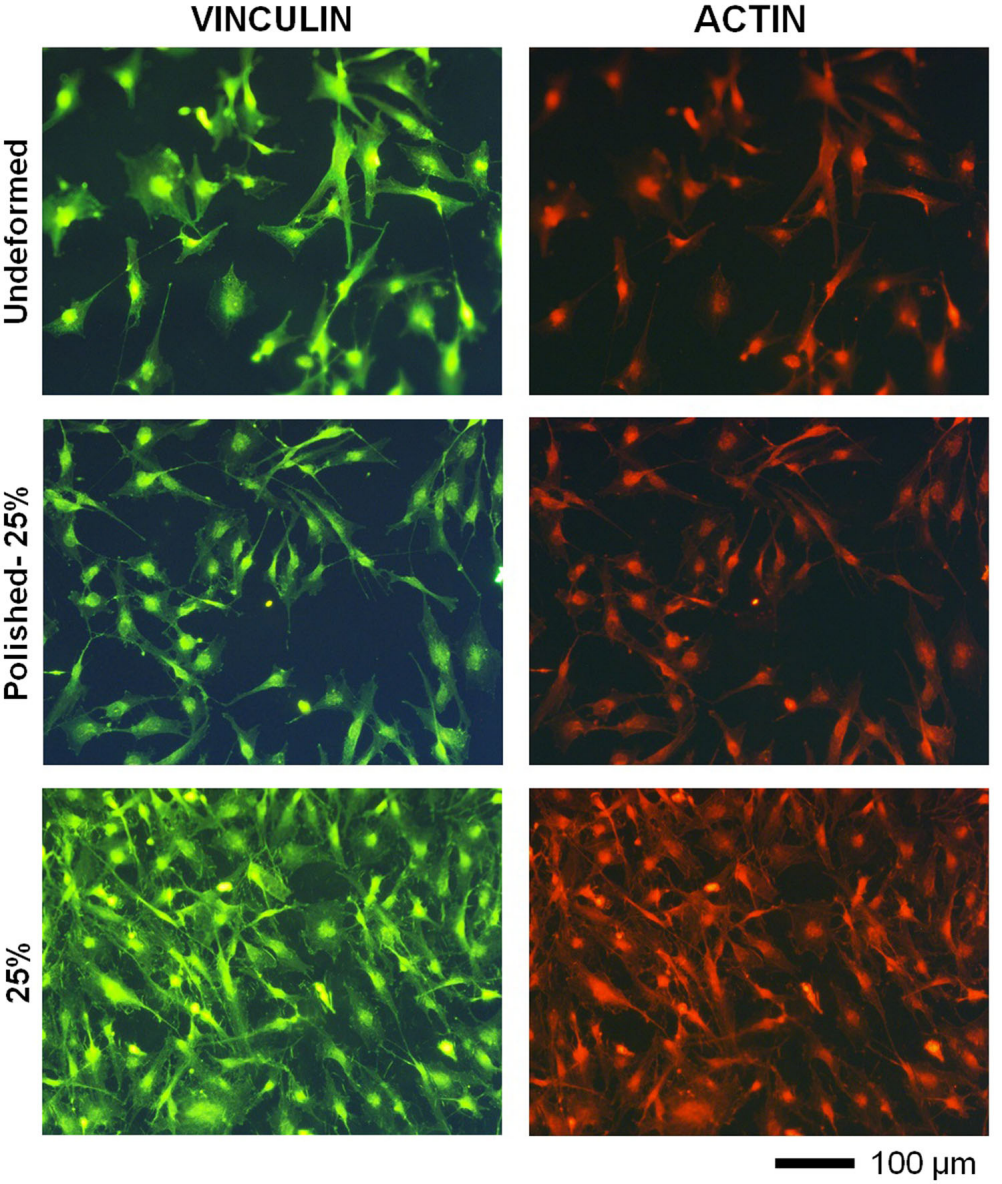
Immunofluorescence images of the cells attached on undeformed and 25% samples present the influence of surface topography and microstructure on cell response. Greater number of cell attachment on the 25%-samples compared with the polished-25% sample further suggest that elimination of topographical cues can impair deposition of focal adhesion proteins and decrease cell attachment

**Fig. 6:**
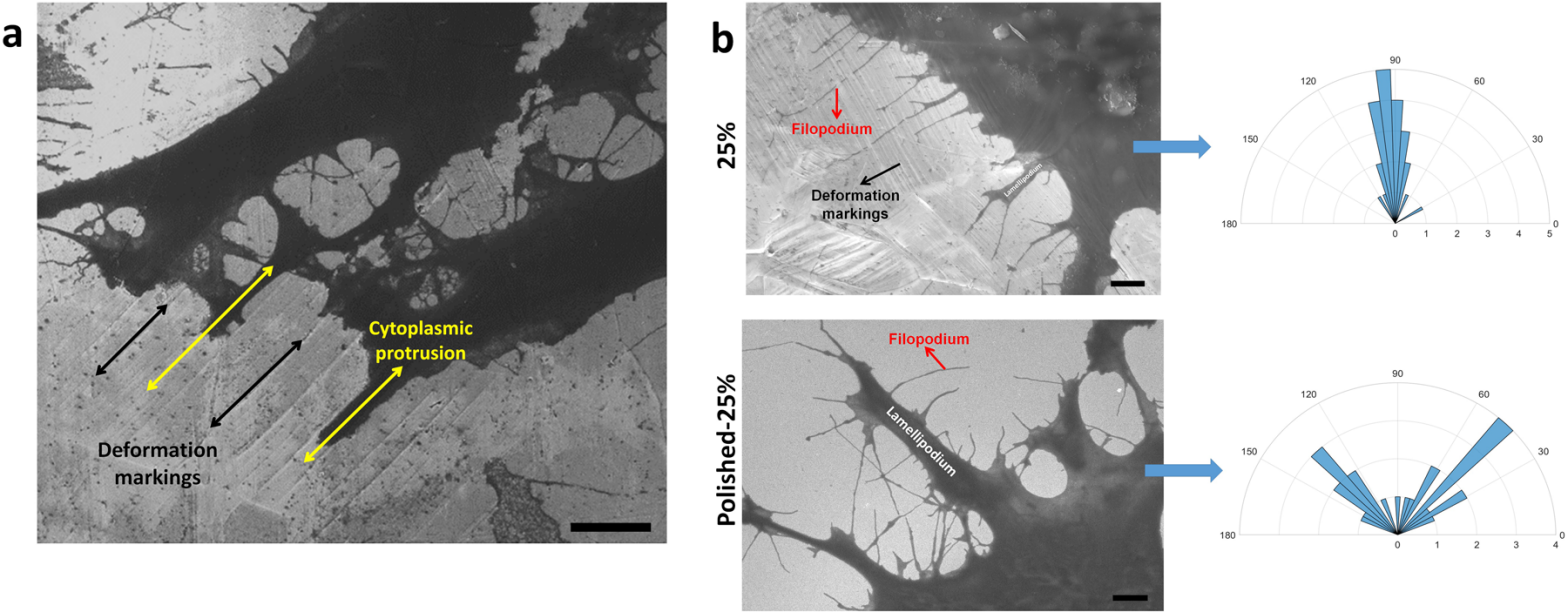
SEM images of cytoplasmic protrusions show dependency on the length scale of deformation markings. **a** Oriented elongation of cytoplasmic protrusions parallel to microscale deformation markings is observed on 5% deformed sample. Scale bar, 5 μm. **b** SEM images of protrusions on the 25% sample and the polished-25% samples show the influence of topography and microstructure on filopodia orientations. Angular histograms presents an aligned elongation of filopodia on 25% sample in the direction predominantly about 90°, whereas filopodia on polished-25% samples elongate in random directions with a larger range of angle. Scale bars, 3 μm

**Fig. 7:**
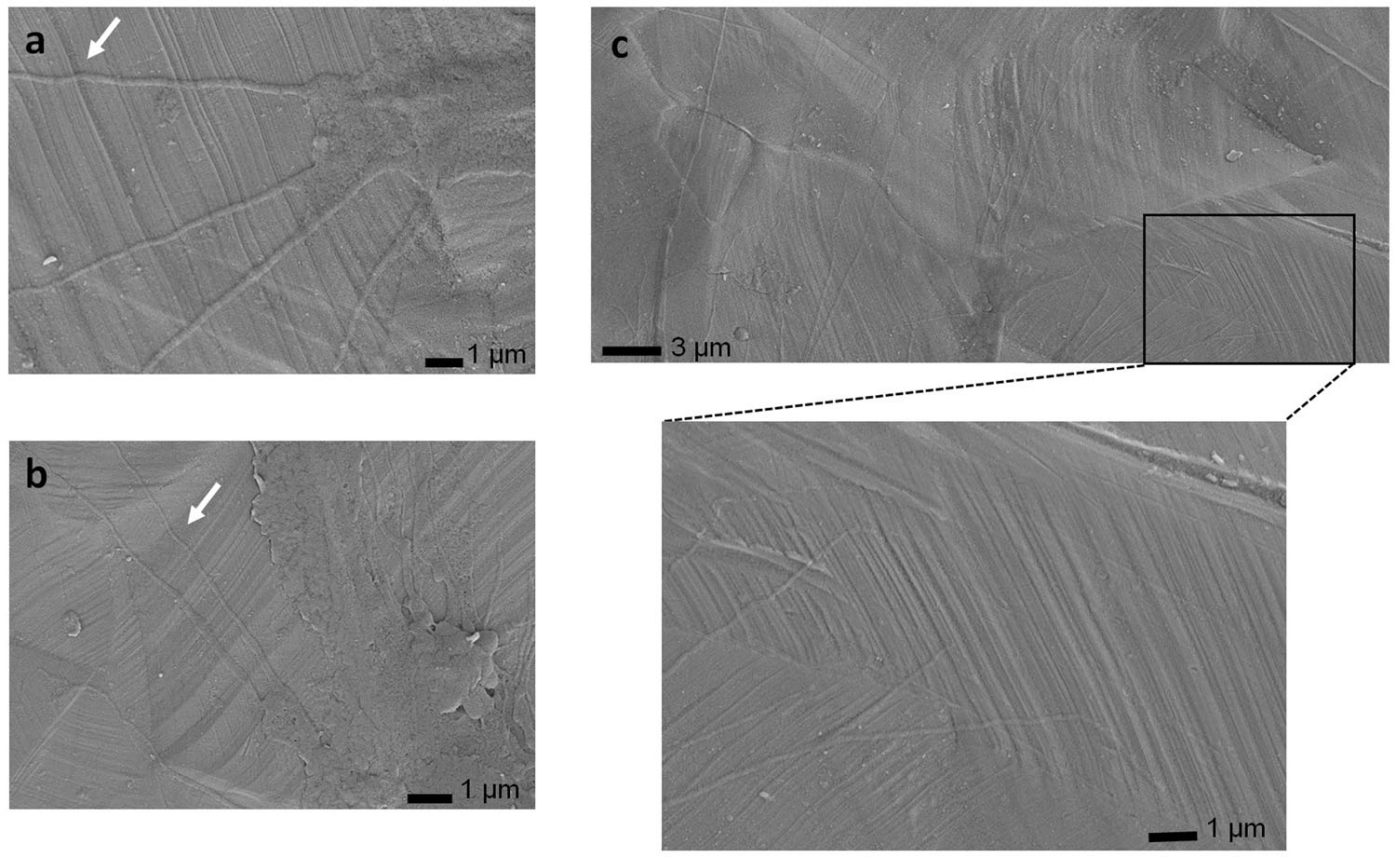
SEM images of the filopodia elongated along the deformation markings on the 25% sample. **a**, **b** Kinkings of filopodia pointed out with white arrows indicate large contact forces between submicro-scale deformation markings and filopodia. **c** Filopodia elongates via sensing submicro-scale topographical cues. (Figure is more readable and visible on the web version rather than in print)

Adhesion behavior and morphology of GBMs on the undeformed and post-mortem samples was analyzed via fluorescence microscope (Fig. 5) and SEM (Figs. 6 and 7). Number of cell attachment on 25% samples normalized by that of the undeformed sample showed 1.7-fold increase. Cell attachment on polished-25% sample also showed 1.2-fold increase. Non-parametric Kruskal–Wallis test showed that mean cell attachment values of each group are significantly different from other (*p* < 0.001). It is important to note, based on one-way ANOVA and following multiple comparisons (LSD), that mean values for cell attachment on the undeformed and polished-25% samples was not significantly different (*p* = 0. 064).

Next, cell elongation has been investigated and cellular aspect ratios (AR) on undeformed, polished-25 and 25% deformed samples were measured as 1.83 ± 0.20, 1.81 ± 0.20 and 2.67 ± 0.36 respectively. Non-parametric Kruskal−Wallis test indicate that means of the groups are significantly different (*p* < 0.001). One-way ANOVA and following multiple comparisons (LSD) show cell elongation on undeformed and polished-25% samples was not significantly different (*p* = 0. 887), whereas AR of 25% sample showed significant difference from others.

Finally, cytoplasmic protrusions have been investigated in detail via SEM, and Fig. 6a presents GBM attachment on the 5% sample. A closer investigation of the figure suggests that the directional elongation of microscale cytoplasmic protrusions can be observed along the grooves formed by deformation markings in microscale; which, to the best of this author’s knowledge, is first time ever observed for GBMs on a metallic material. Interestingly, however, on the 25% sample, elongation of cytoplasmic protrusions along the submicro-sized grooves could not be observed. However, filopodia (submicro-scaled protrusions) on the 25% sample present organized elongation in a direction that is almost perpendicular with respect to deformation markings (Fig. 6b). Interactions of these submicro-scale deformation markings and filopodia were further analyzed via SEM. Elongated filopodia elongation was captured traversing over submicro-scale deformation markings exploring for topographical signals (Fig. 7), which plays crucial role in cancer cells since cell movement is controlled by these cytoplasmic protrusions. Furthermore, kinking of filopodia was observed on this sample (Fig. 7a, b), evidencing constraint induced by deformation markings and contact forces applied on filopodium at the interaction points with the surface.

It is important to note at this point that filopodia on the polished-25% sample did present unorganized and random direction (Fig. 6b). Angular histogram of filopodia orientation on these two samples presents this randomness clearly, where elongation of filopodia on 25% sample presents localization around 90° (Fig. 6b).

## Discussions

In this research, evolution of microstructural and mechanical properties of 304 stainless steels upon plastic deformation is analyzed, and their relationship with contact guidance of GBM cells are reported. The mechanical twins and/or martensite platelets observed on the undeformed sample (Fig. 1a) most possibly stems from the manufacturing of the sheet metal, where annealing upon rolling process was not able to fully recover the plastic deformation mechanisms [18]. It is noteworthy that these markings have localized in certain regions with low volume fractions. As a result, amount of martensite stays below the detection limit of XRD and this sample presents fully austenite peaks in its XRD spectra (Fig. 2). Upon plastic deformation the volume fraction of martensite increases [11], which can result with enhanced cell response on deformed metallic biomaterials [23].

Increased volume fraction of deformation markings on 25% sample (Fig. 1e) stems from excessive load applied on the material throughout deformation, which was accommodated through the activation of strengthening mechanisms. Furthermore, curvatures observed in the deformation markings of 25% sample (Fig. 1e), which result from inhomogeneous strain within the grains, indicate in-grain lattice misorientation [7]. This consequently can inhibit the formation of new deformation twins and influence hardening response of the material [7]. In addition, micro-crack observed on 25% sample upon plastic deformation can also be initiated during manufacturing of the implants and lead to diminishing of corrosion resistance [44] and fracture of the material during service, necessitating surgical interventions.

SEM observations of the deformation markings and their corresponding effect on surface topography present directional orientation on the implant surface (Fig. 1c, d, f), enhancing the anisotropy of the material. Moreover, in parallel with plastic straining, spacing of deformation markings decrease from microscale (Fig. 1c, d) down to submicro-scale (Fig. 1f). Specifically, the line profile traversing over the deformation markings presents the microsized grooves on the 5% sample (Fig. 3c) and submicro-sized grooves achieved on the 25% sample (Fig. 3f). These grooves constitute significance, since they can bio-mimic the characteristics of white matter tracts, which are major GBM migration highways [31]. Furthermore, they can also mimic anisotropic ECM fibers and provide tracks for invading cells [45]. Therefore, obtaining directional micro- or submicro-scaled textures on material surfaces could provide insights on the invasion pathways of GBM, which would lead to the development of enhanced therapeutic tools targeting invasion mechanisms. It should be noted that, although similar groove-like topographies can also be created via lithography or femtosecond laser techniques [46–48], simple tension or compression tests provide an easier way to form 3D anisotropic microstructure and topography via surface relief [2, 20, 21].

The effect of microstructural evolution on mechanical response is also investigated. Four-staged hardening response of the 25% sample (Fig. 4a) shows similarity with coarse-grained low SFE FCC metals:[7, 14, 43] (1) Decline of SHR observed in stage I might stem from planar dislocation activity [4]. (2) The increase of SHR in stage II is possibly due to formation of deformation twinning [4, 5]. In this stage prevention of cross slip can cause early onset of twinning, leading to further hardening of material [39]. In parallel with straining, grain size gets smaller as twins fragment the grains into smaller grains with different crystallographic orientations [9, 49]. The fragmented grains and twin boundaries provide more barriers hindering dislocation motion, and lead to increasing SHR at this stage of deformation [50]. (3) Next, SHR decrease is observed in stage III, which might stem from difficulty of new twin formation [6, 7]. (4) However, throughout stage IV, continuing interaction of twinning, slip, and martensite keeps hardening rate at a constant level around for a large strain range. Overall, the activation and interaction of the aforementioned mechanisms led the material to have high strength and ductility, in parallel with providing regions with high surface energy, which facilitated the deposition of focal adhesion proteins and eased cell attachment [2]. The evolution of mechanical properties of the samples was further analyzed with nanoindentation test (Fig. 4b). The shift towards lower indentation depths observed in the load versus depth to indentation curves of the samples suggests that surface hardness increases in concomitant with work hardening, thereby enhancing resistance against the motion of indenter.

Adhesion response and morphology of GBMs on the deformed samples has been analyzed via SEM and fluorescence microscope. The greatest number of cell attachment observed on 25% sample (Fig. 5) could be stemming from enhanced surface roughness, wettability, and free energy of this sample by virtue plastic deformation [22]. Specifically, greater density of strengthening mechanisms or twin and grain boundaries can provide high-energy regions promoting deposition of focal adhesion proteins. Recently, a similar finding was reported with breast cancer cells, where the idea of using metallic biomaterials as potential therapeutic tools for capturing cancer cell was proposed. Similar results obtained in this study can suggest that material with modulated surface topography and microstructure could be utilized to attract GBMs on the surface and disable invasion capability. In addition, greater number of cell attachment observed on 25% samples with respect to the polished-25% sample (Fig. 5) further suggests that elimination of topographical cues can impair deposition of focal adhesion proteins and result with decreased cell attachment. These findings indicate that plastic deformation induced surface topography can play an essential role in modulating adhesion and proliferation of GBMs.

Morphological changes and greater AR of GBMs observed on 25% sample could give insight on tendency for invasion. Elongated cells can indicate that GBMs on 25% sample shows more mesenchymal cell characteristics implying a more invasive tumor phenotype [51]. On the other hand, GBMs attached on mirror polished undeformed and polished-25% samples could not exploit topographical cues to regulate morphology and invasion behavior. This shows that extracellular physical factors could play crucial role in determining intracellular structure and consequently invasiveness of cancer cells. However, it is important to point out that greater number of cells attached on the 25% samples may have confined the cells in a narrower region and increase intercellular interactions. As a result this might have affected the elongation behavior on 25% sample. Therefore, observations with much less number of cells are needed, where the effect of intercellular interactions on cell behavior can be eliminated.

Interaction of deformation markings with cytoplasmic protrusions was closely investigated since they play critical role at leading edges of invading cancer cells. The alignment of microscale protrusions parallel to the microsized grooves on 5% sample (Fig. 6a) suggests that, the directional patterns created via surface relief can be facilitated in contact guidance of GBMs. On the other hand, the fact that the microscale cytoplasmic protrusions parallel to the grooves was not observed on the 25% deformed sample might be related with the spacing of the deformation markings. That is, straining from 5 to 25% leads to increase in the volume fraction of deformation markings within a grain and also results in diminished spacing between each line down to submicro-scale. Similarly, studies carried out on osteoblast cells have shown the decisive effect of the groove width on cell response [20, 47]. These findings imply that contact guidance of micro-scale cytoplasmic protrusions GBM can be promoted along micro-scaled grooves formed with a lower degree of plastic deformation.

Next, filopodia adhesion was analyzed on the 25% deformed samples via SEM. Preferred elongation of GBM filopodia along the direction almost perpendicular to the submicro-scale surface markings (Fig. 6b) was observed for the first time in literature. Such that, these deformation markings seem to enable elongation of filopodia via providing adhesion sites for focal contact points along the ridges of the texture. As a result of these physical interactions filopodia bending/kinking at these contact points was captured (Fig. 7a, b). This can imply that contact forces between the deformation markings and filopodia can be large enough to control alignment of these filaments. Observed structure suggests that submicro-scale deformation markings can act as an obstacle to directional elongation of filopodia. This finding could be important because in migrating cells filopodia probe the environment for a clue at the leading edge of the cell through dynamic contractions and retractions [28, 52]. Hence, this behavior can provide important insight on understanding the role of GBM filopodia in substrate exploring and determining invasion pathways. It should be noted that, the filopodia on the polished-25% sample presented unorganized and oblique adhesion (Fig. 6b). This finding further suggests that filopodia elongation can be modulated by topographical and microstructural cues and understanding the mechanisms controlling their interaction could be utilized to impair cancer invasion. However, in order to obtain much solid evidence further research is necessitated, where time-lapse imaging and theoretical modeling could also be implemented.

It is also noteworthy that, the elongation over the grooves on 25% sample may lead to fragmented focal adhesion protein expression [53], which stems from the nature of topography of the deformation markings with grooved and ridged structures. This may consequently cause weaker cell adhesion, resulting in easy and rapid retraction of filopodia [53], which may affect the migration activity of the cancer cells. Hence, rapid retraction of the filopodia elongated perpendicular to the deformation markings can help cancer cells to get more information in a shorter period of time to take action for directional movement. It is important to note that, filopodia did not present a similar adhesion on the 5% sample, which might be due to the larger spacing of the deformation markings, resulting a larger gap between the grooves.

It is should be noted that, interaction of ECM proteins with deformation markings also warrants investigation, since they have essential role in contact guidance [54, 55] and migration of cancer cells [45]. Specifically, elucidating the molecular interactions underlying ECM-cell interactions on these textured surfaces can provide important findings. Recently, abnormal elongation of collagen matrices orthogonal to cell alignment was observed, which was explained with formation of focal adhesion points along nanoscaled-grooves [56]. This finding could indicate that elucidating the orientation of ECM proteins could further increase one’s knowledge to better understand the adhesion and invasion behavior of cancer cells.

## Conclusion

The current study was carried out with the motivation to elucidate the relationship between the evolution of microstructural and mechanical properties of the metallic biomaterials, and glioblastoma cell (GBM) response. Anisotropic deformation markings activated on the 304 stainless steel samples subjected to tensile test up to 5 and 25% of strain levels led to alignment of cytoplasmic protrusions, which to the best of author’s knowledge was first time observed on a metallic biomaterial. Observed elongation of filopodia almost perpendicular to submicro-scale deformation markings and kinking at contact points can provide critical insights on to how cancer cells sense their microenvironment and take action for invasion. These findings suggest that cytoplasmic protrusions of GBMs in different length scales can be regulated via varying degrees of plastic deformation and volume fractions of deformation markings. Based on these findings, implants for therapeutic purposes can be designed, which target migration pathways of GBMs and regulate capability of invasion.
